# Isolation of Pro-Osteogenic Compounds from *Euptelea polyandra* That Reciprocally Regulate Osteoblast and Osteoclast Differentiation

**DOI:** 10.3390/ijms242417479

**Published:** 2023-12-14

**Authors:** Ryuichiro Suzuki, Yoshiaki Shirataki, Akito Tomomura, Kenjiro Bandow, Hiroshi Sakagami, Mineko Tomomura

**Affiliations:** 1 Department of Pharmaceutical Sciences, Faculty of Pharmacy and Pharmaceutical Sciences, Josai University, 1-1 Keyakidai, Sakado 350-0295, Saitama, Japan; 2 Division of Biochemistry, Department of Oral Biology & Tissue Engineering, Meikai University School of Dentistry, Sakado 350-0283, Saitama, Japankbando@dent.meikai.ac.jp (K.B.); 3 Meikai University Research Institute of Odontology (M-RIO), Sakado 350-0283, Saitama, Japan; sakagami@dent.meikai.ac.jp

**Keywords:** *Euptelea polyandra* Sieb. et Zucc, isoquercitrin, astragalin, osteoblast, osteoclast, osteoporosis

## Abstract

Plants contain a large number of small-molecule compounds that are useful for targeting human health and in drug discovery. Healthy bone metabolism depends on the balance between bone-forming osteoblast activity and bone-resorbing osteoclast activity. In an ongoing study searching for 22 plant extracts effective against osteoporosis, we found that the crude extract of *Euptelea polyandra* Sieb. et Zucc (*E. polyandra*) had osteogenic bioactivity. In this study, we isolated two compounds, isoquercitrin (**1**) and astragalin (**2**), responsible for osteogenic bioactivity in osteoblastic MC3T3-E1 cells from the leaf of *E. polyandra* using column chromatography and the spectroscopic technique. This is the first report to isolate astragalin from *E. polyandra*. Compounds (**1**) and (**2**) promoted osteoblast differentiation by increasing alkaline phosphatase (ALP) activity and alizarin red S stain-positive calcium deposition, while simultaneously suppressing tartrate-resistant acid phosphatase (TRAP)-positive osteoclast differentiation in RAW264.7 cells at non-cytotoxic concentrations. Isoquercitrin (**1**) and astragalin (**2**) increased the expression of osteoblastic differentiation genes, Osterix, ALP, and Osteoprotegerin in the MC3T3-E1 cells, while suppressing osteoclast differentiation genes, TRAP, Cathepsin K, and MMP 9 in the RAW264.7 cells. These compounds may be ideal targets for the treatment of osteoporosis due to their dual function of promoting bone formation and inhibiting bone resorption.

## 1. Introduction

Bone mineralization is controlled by a dynamic and balanced mechanism, with bone formation mediated by osteoblasts and bone resorption mediated by osteoclasts. However, under the dysregulated condition of bone metabolism, especially in menopausal women, the lack of estrogen leads to increased bone resorption that results in osteoporosis [1]. Osteoporosis is a metabolic bone disorder characterized by reduced bone mass and a weak bone microarchitecture, leading to a susceptibility to bone fracture. It is a critical public health problem worldwide, affecting more than 200 million individuals, and the number of osteoporosis patients is expected to increase in an aging society [2,3]. Bisphosphonates are well-known antiresorptive drugs that target osteoclasts to treat osteoporosis. However, bisphosphonates reduce bone remodeling. As a side effect, they induce osteonecrosis of the jaws [4]. It is therefore desirable to develop safe therapeutic agents that not only suppress bone resorption, but also have a beneficial effect on bone formation.

The maturation and function of osteoblasts and osteoclasts in bone remodeling are tightly regulated, and are influenced by various factors including estrogen, the receptor activator of nuclear factor κ-B ligand (RANKL), osteoprotegerin (OPG), and the proinflammatory cytokines tumor necrosis factor (TNF)-α, interleukin (IL)-1, and IL-6. The osteoclast is differentiated from monocyte–macrophage lineage by RANKL stimulation, expresses tartrate-resistant acid phosphatase (TRAP), and forms a multinucleated mature large cell to resorb bone matrix. Osteoblasts differentiate from mesenchymal stem cells and express bone matrix proteins and alkaline phosphatase (ALP) for mineral deposition. Osteoblasts regulate osteoclast differentiation and maturation by secreting RANKL, while also secreting OPG to inhibit osteoclast RANKL signaling [5]. Estrogen promotes OPG secretion from osteoblasts and inhibits the secretion of RANKL and proinflammatory cytokines to suppress osteoclastic bone resorption [6,7]. Estrogen replacement therapy is the most effective treatment for estrogen deficiency in menopausal osteoporosis, although it is associated with a risk of endometrial and breast cancer and cardiovascular disease [8].

Natural plant metabolites are used in the treatment of osteoporosis, where polyphenols regulate bone metabolism [9]. Phytoestrogens, plant-derived estrogen-like isoflavones including daidzein, genistein, equol, and ipriflavone, promote bone metabolism [10,11,12,13]. Flavonols including quercetin, myricetin, kaempferol, icariin, and flavanols including epicatechin and epigallocatechin, also promote bone metabolism [14,15,16,17]. *Euptelea polyandra* Sieb. et Zucc (*E. polyandra*) is a tree endemic to Japan [18], and its leaves are used in traditional Japanese health drinks. Terpenoids and saponins isolated from the leaves of *E. polyandra* have antiviral and gastroprotective functions [19,20,21,22,23,24]. However, the effect of *E. polyandra* on bone metabolism has not been elucidated. In the course of our studies in search of bone metabolism regulators from natural plants, we found that the crude leaf extract of *E. polyandra* promotes differentiation in osteoblasts and inhibits differentiation in osteoclasts [25].

In the present study, we isolated two bioactive compounds, isoquercitrin (**1**) and astragalin (**2**), from the leaves of *E. polyandra*. Each of them has dual activities, promoting osteoblast differentiation and inhibiting osteoclast differentiation.

## 2. Results

### 2.1. Isolation of Osteoblast Differentiation-Promoting Compounds from E. polyandra

**Figure 1:**
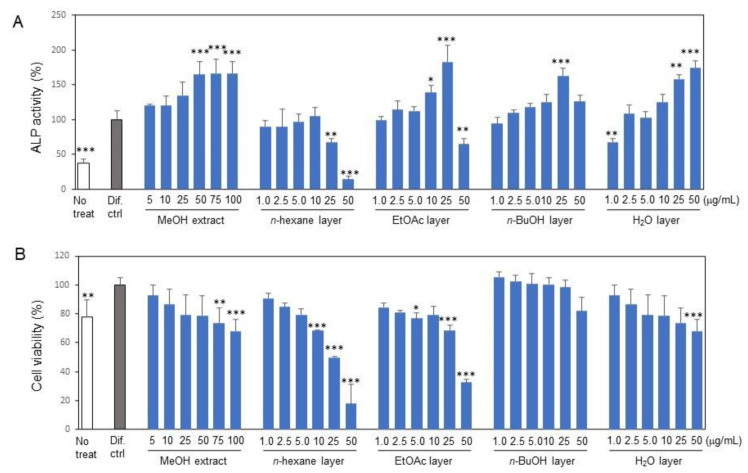
Effects of methanol (MeOH) extract and subsequent fractions from the extract on the alkaline phosphatase (ALP) activity and viability of MC3T3-E1 osteoblasts. Cells were seeded in 96-well plates and, at subconfluent culture, ascorbic acid and β-glycerophosphate were added to the samples (differentiation condition: Dif ctrl). (**A**) ALP activity and (**B**) cell viability of the cells treated with the indicated doses of MeOH extract, and subsequent fractions of *n*-hexane, ethyl acetate (EtOAc), *n*-butanol (*n*-BuOH), and H_2_O treatments. Data are presented as the mean ± standard deviation. * *p* < 0.05, ** *p* < 0.01, and *** *p* < 0.001 versus differentiation control.

In a previous study, we found that MeOH extract from *E. polyandra* leaves promoted osteoblast differentiation [25]. In this study, we aimed to isolate substances that stimulate osteoblast activities from the leaves of *E. polyandra*. First, we extracted the compounds from the dried leaves of *E. polyandra* using MeOH and subsequently separated them using *n*-hexane, EtOAc, *n*-BuOH, and H_2_O layers (Supplemental Scheme S1). The ALP activity of MC3T3-E1 cells increased 2.5-fold after 7 days of culture in the differentiation medium when compared to the undifferentiated culture. Treatment with the MeOH extract of *E. polyandra* dose-dependently increased the ALP activity by about 1.7 times compared to the differentiated control (Figure 1A). Treatment with the EtOAc, *n*-BuOH, and H_2_O fractions enhanced the ALP activity, while the *n*-hexane fraction did not enhance the ALP activity and showed higher cytotoxic activity (Figure 1B). We selected the EtOAc fraction for the next step of purification because it showed the highest ALP activity, retaining an activity comparable to that of the MeOH extract, and the activity per weight was increased from 50~100 μg/mL to 25 μg/mL.

**Figure 2:**
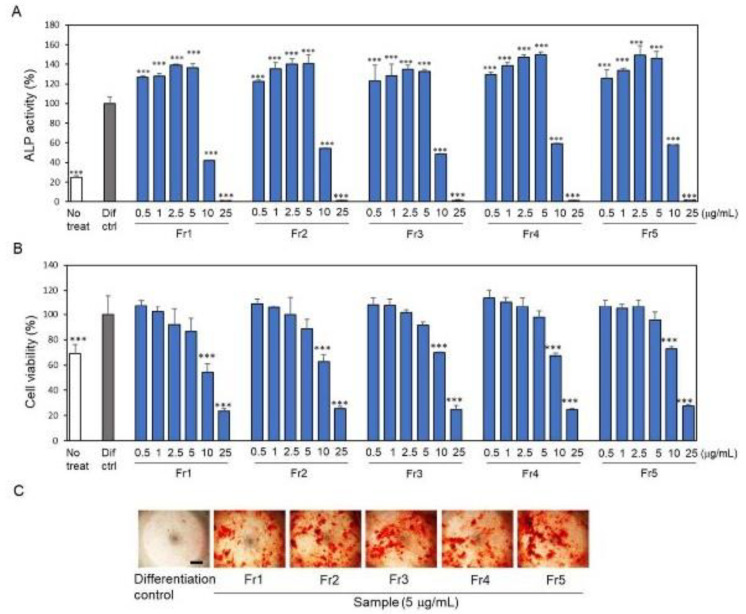
Effects of column-separated fractions of ethyl acetate (EtOAc) layer on alkaline phosphatase (ALP) activity, alizarin red S staining, and cytotoxicity of MC3T3-E1 osteoblasts. Five fractions (Fr1: 50% MeOH, Fr2: 75% MeOH, Fr3: 100% MeOH, Fr4: 50% MeOH/50% Acetone, Fr5: 100% Acetone) were obtained from the EtOAc layer of the MeOH extract by column separation. (**A**) ALP activity and (**B**) cytotoxicity of MC3T3-E1 cells were measured after 7 days of culture, and (**C**) alizarin red S staining was performed after 14 days of culture in a differentiation medium containing ascorbic acid and β-glycerophosphate. Bar, 500 μm. Data are presented as the mean ± standard deviation. *** *p* < 0.001 versus differentiation control (Dif ctrl).

Column chromatography and preparative HPLC were subsequently performed to isolate the osteoblast-differentiating compounds from the bioactive and high-yield EtOAc fraction (Supplemental Scheme S2). The EtOAc fraction (4.2 g) was applied to an ODS column and sequentially eluted with 50% MeOH (Fr1), 75% MeOH (Fr2), 100% MeOH (Fr3), 50% MeOH-50% Acetone (Fr4), and 100% Acetone (Fr5). All fractions increased the ALP activity up to 5 μg/mL in a dose-dependent manner without cytotoxicity (Figure 2A,B). Furthermore, alizarin red S staining showed that all fractions also increased mineralized nodule formation, a late-phase marker of osteoblast differentiation (Figure 2C). Among these fractions, none of them had a particularly high activity compared to the others. The EtOAc fraction is thought to contain several bioactive compounds that stimulate the differentiation of osteoblasts.

**Figure 3:**
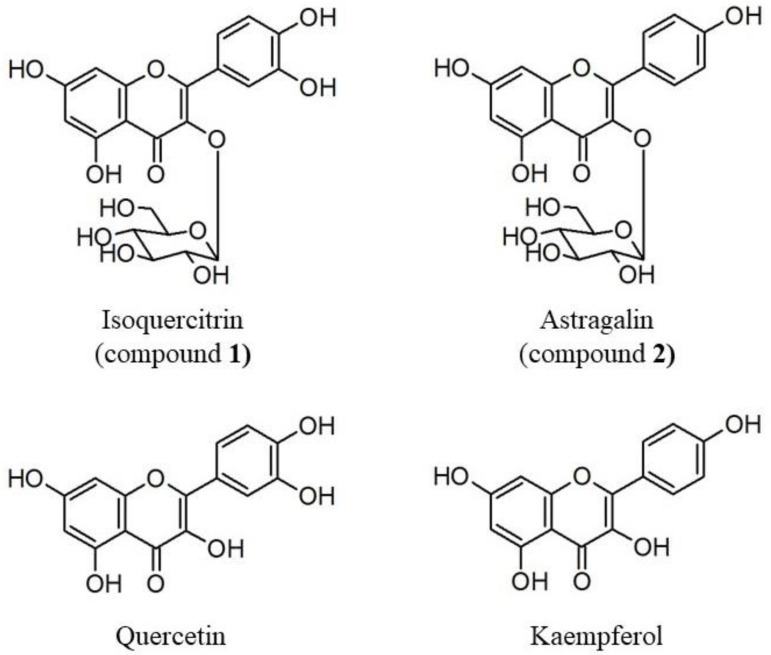
Structures of compounds isolated from *Euptelea polyandra* and each aglycone.

Finally, an aliquot (170 mg) of the most abundant 50% MeOH eluate (Fr1) was subjected to medium-pressure liquid chromatography (MPLC) fractionation and thin-layer chromatography (TLC) analysis. Among the 18 fractions, fractions No. 8 and No. 11 showed a single spot on the TLC plate. Using nuclear magnetic resonance (NMR) analysis, two compounds were identified as quercetin 3-*O*-β-D-glucopyranoside (compound **1**: isoquercitrin; 25.8 mg) and kaempferol 3-*O*-β-D-glucopyranoside (compound **2**: astragalin; 18.9 mg) (Figure 3 and Supplemental File S1) [26,27].

**Figure 4:**
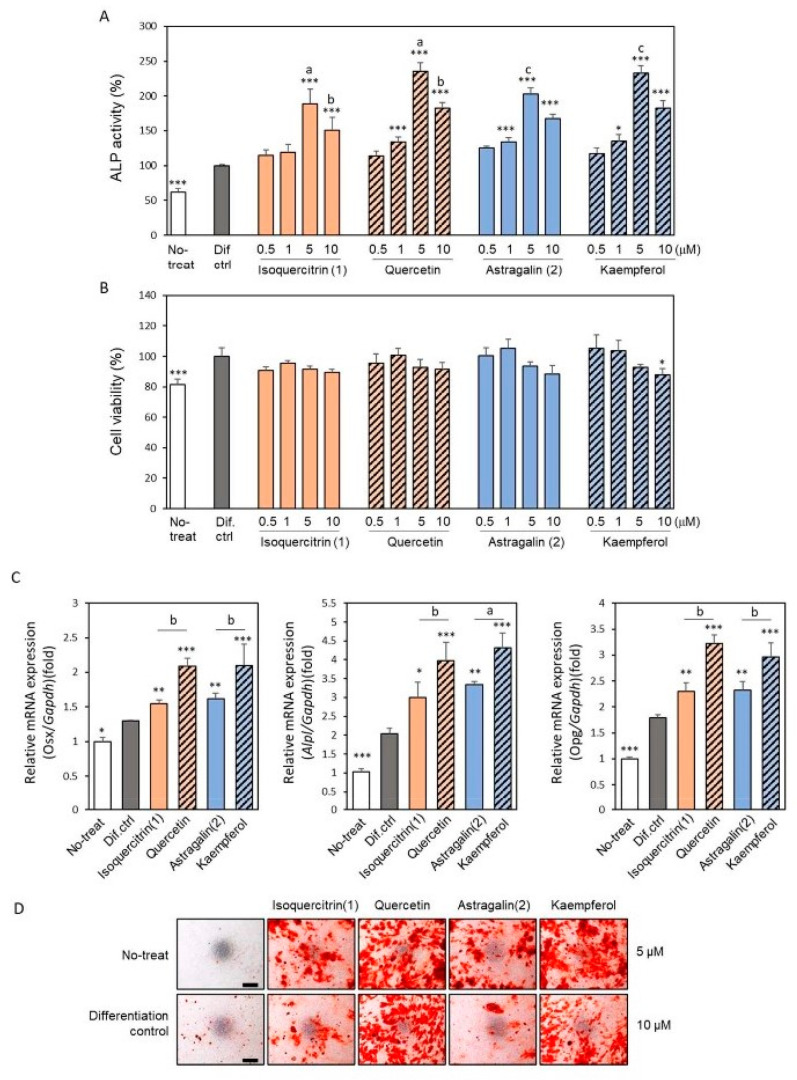
Effect of isolated compounds and their aglycones on the differentiation within MC3T3-E1 cells. Compound **1** (isoquercitrin) and compound **2** (astragalin) and each of the aglycones, quercetin and kaempferol, were added along with ascorbic acid and β-glycerophosphate to subconfluent-cultured MC3T3-E1 cells for osteoblast differentiation. (**A**) ALP activity and (**B**) cell viability assays were measured after 7 days of culture. (**C**) Expression levels of mRNA of *Osx* (day 2), *Alp* and *Opg* (day 4) were analyzed by RT-qPCR. (**D**) Alizarin red S staining was performed at day 14 of culture after sample addition. Bar, 500 μm. Data are presented as the mean ± standard deviation. * *p* < 0.05, ** *p* < 0.01 and *** *p* < 0.001 versus differentiation control. Small letters indicate significance between the same dose of compound and its aglycone (^a^ *p* < 0.001; ^b^ *p* < 0.01; ^c^ *p* < 0.05).

The effects of isolated compounds on the ascorbic acid/β-glycerophosphate-induced osteoblast differentiation in MC3T3-E1 cells were further investigated (Figure 4).

Analysis of the ALP activity and gene expressions of molecular biomarkers for osteoblast and alizarin red S staining was performed on osteoblasts treated with isoquercitrin, astragalin, and their respective aglycones, quercetin and kaempferol. Isoquercitrin and astragalin significantly promoted the ALP activity of MC3T3-E1 cells after a 7-day differentiation induction. The induction fold was a little lower than for the respective aglycon (Figure 4A). There was no significance between compounds **1** and **2**. The cytotoxicity of isoquercitrin, astragalin, and their aglycones quercetin and kaempferol was minimal (Figure 4B). Reverse transcription-quantitative PCR (RT-qPCR) analysis revealed that the increased ALP activity was associated with an increase in ALP mRNA expression (Figure 4C). Other molecular biomarkers, Osterix and OPG, which are related to osteoblast differentiation and maturation, were investigated [5,28]. Osterix is known as master transcription factor, as is Runt related transcription factor 2 (Runx2) and OPG is a key negative regulator of osteoclastogenesis and secreted from osteoblasts. Both mRNA expressions were also upregulated in the treatment with isoquercitrin, astrogalin, and their respective aglycones compared with the untreated differentiated control cells. Consistent with the result of ALP activity, aglycons yielded more significant increases in the expression of these genes. At 14 day of culture, mineralization was assessed with alizarin red S staining. Isoquercitrin and astragalin significantly increased the mineralization of MC3T3-E1 cells but to a lesser extent than quercetin and kaempferol (Figure 4D).

### 2.2. Effects of the Isolated Compounds on RANKL-Induced Osteoclastogenesis

**Figure 5:**
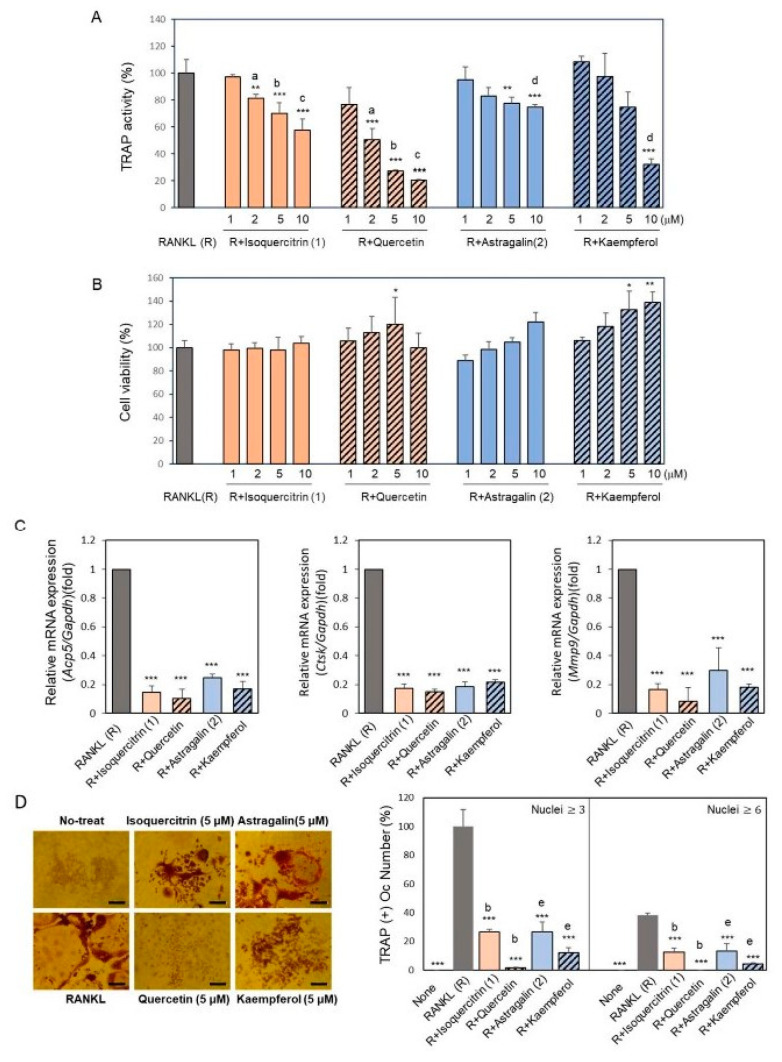
Effect of isolated compounds and their aglycones on tartrate-resistant acid phosphatase (TRAP) activity, TRAP staining, and cell viability of RAW264.7 cells. Isoquercitrin (**1**) and astragalin (**2**) and their aglycones, quercetin and kaempferol, were added with RANKL to cultured RAW264.7 cells after 24 h and further cultured for 3~4 days. (**A**) TRAP activity and (**B**) cell viability. (**C**) Expression levels of mRNA of *Acp5*, *Ctsk*, and *Mmp9* were analyzed by RT-qPCR after 3 days of induction. (**D**) TRAP-staining. TRAP-positive cells containing three or more nuclei were counted as multinuclear cells (MNCs). Bar, 100 μm. Data are presented as the mean ± standard deviation. An asterisk indicates the significance of RANKL treatment. * *p* < 0.05, ** *p* < 0.01, *** *p* < 0.01 vs. RANKL-treated differentiated control. Small letters indicate a significance between the same dose of a compound and its aglycone (^a^ *p* < 0.01; ^b^ *p* < 0.001; ^c^ *p* < 0.001; ^d^ *p* < 0.001; ^e^ *p* < 0.05).

To further investigate the role of isoquercitrin (**1**) and astragalin (**2**) together with each of the aglycones, quercetin and kaempferol, in bone remodeling, we demonstrated their effects on RANKL-induced osteoclastogenesis using the macrophage lineage cell line RAW264.7 cells by monitoring TRAP activity. The compounds isoquercitrin (**1**) and astragalin (**2**) suppressed the TRAP activity in a dose-dependent manner, whereas the aglycones, quercetin and kaempferol, inhibited it more significantly (Figure 5A). Furthermore, the anti-osteoclastic activity was stronger in quercetin and isoquercitrin than in kaempferol and astragalin. These compounds did not suppress the cell viability, indicating that their anti-osteoclastic activity was not associated with their cytotoxic effect (Figure 5B). We also confirmed the effects of these compounds on the gene expression of osteoclast molecular biomarkers such as cathepsin K (*Ctsk*) and matrix metalloproteinase 9 (*Mmp9*), in addition to tartrate-resistant acid phosphatase type 5 (*Acp5*), which are important for the functional osteoclast phenotype involved in bone resorption [29] (Figure 5C). The RANKL-induced mRNA expression of those genes was significantly inhibited by treatment with compounds (**1**), (**2**), and their respective aglycons. The inhibitory effects tended to be stronger for the aglycones than for the glycosides. TRAP staining showed that the formation of TRAP-positive multi-nucleated osteoclasts was also suppressed by isoquercitrin and astragalin and their aglycones, quercetin and kaempferol (Figure 5D).

## 3. Discussion

Natural plant metabolites have become an important source for the discovery of new drugs. After an initial screening in search of plant extracts effective for the treatment of osteoporosis, we found that the crude extract of *E. polyandra* showed osteogenic bioactivity. In this study, we isolated two flavonols responsible for osteogenic bioactivity, isoquercitrin and astragalin, from the leaves of *E. polyandra*. This is the first study to report the isolation of astragalin from *E. polyandra*. Both of these flavonols promoted osteoblast differentiation and suppressed osteoclast differentiation in a non-cytotoxic manner (Figure 4 and Figure 5). These compounds could be ideal drug targets for the treatment of osteoporosis because they exhibit the dual function of promoting bone formation and inhibiting bone resorption. Indeed, it has been reported that isoquercitrin improves bone characteristics and bone turnover in ovariectomized rats [30], and its involvement in osteoblastic differentiation has been demonstrated [31]. Astragalin also promotes bone formation in ovariectomized-induced osteoporotic mice [32], and increases osteoblast activity while decreasing osteoclast activity [33].

The structural difference between isoquercitrin and astragalin is the presence or absence of a hydroxyl group at the 3′ position (Figure 3), but no significant difference was observed in terms of promoting osteoblast differentiation (Figure 4). However, the inhibition of osteoclast differentiation is higher in isoquercitrin than in astragalin, suggesting that the structure–activity relationship is modulated in these cell types (Figure 5). Isoquercitrin and astragalin are glycosides of quercetin and kaempferol, respectively. The bioactivity of quercetin and/or kaempferol is strong compared to its corresponding glycoside of isoquercitrin and/or astragalin, in terms of its promotion of osteoblast differentiation and inhibition of osteoclast differentiation. Therefore, it is possible to deduce that the addition of glycan to quercetin and/or kaempferol weakens their bioactivity in the culture cells used in this study. Regarding quercetin and kaempferol, many papers report that they have beneficial effects on bone metabolism, mimicking estrogenic bioactivity [34,35,36,37,38,39].

Estrogen has pleiotropic functions that can simultaneously regulate osteoblasts and osteoclasts in a coordinated manner [40,41]. However, estrogen treatment for osteoporosis increases the risk of breast cancer. Flavonoids are an important group of phytoestrogens. Quercetin and kaempferol have been reported to bind to nuclear estrogen receptors (ERs) and induce osteoblast differentiation [42,43]. The isolated isoquercitrin and astragalin were found to have a lower capacity for inducing osteoblast differentiation than aglycones, suggesting that isoquercitrin and astragalin may be lesser agonists of ERα compared to the aglycones quercetin and kaempferol. Isoquercitrin and astragalin are bound to glucose, which increases their water solubility. It is unclear whether they can be deglycosylated at the cell membrane and pass through it. It has also been reported that the membrane type of the ER plays a role in bone metabolism [44]. It is possible to deduce that isoquercitrin or astragalin binds to the membrane type of ER rather than intracellular ER. Different types of estrogen signaling pathways, not only through ERs, but also through ER-related receptors, have now been reported [45,46]. The differences in the biological effects of the four flavonoids may be due to differences in their respective signaling pathways. The four flavonoids studied in this report may be applicable to the development of new drugs for osteoporosis; however, more detailed structure–function analyses and in vivo efficacy studies are required in the future.

## 4. Materials and Methods

### 4.1. Materials

The dried leaves of *E. polyandra* were used for extraction and fractionation in this study. *E. polyandra* leaves were collected from the botanical garden of Josai University, Saitama, Japan, in May 2016. The plant was authenticated by Dr. Yoshiaki Shirataki. The voucher samples were deposited in the Laboratory of Natural Products and Phytochemistry, Department of Pharmaceutical Sciences, Faculty of Pharmacy and Pharmaceutical Sciences, Josai University. The α-minimum essential medium (α-MEM), ascorbic acid, paraformaldehyde, nitro-blue tetrazolium chloride (NBT), 5-bromo-4-chloro-3′-indolylphosphate *p*-toluidine salt (BCIP), 3-(4,5-dimethyl-2-thiazolyl)-2,5-diphenyl-2*H*-tetrazolium bromide (MTT), sodium dodecyl sulfate (SDS), and dimethyl sulfoxide (DMSO) were purchased from Fujifilm-Wako pure chemical industries (Osaka, Japan). Penicillin and streptomycin were purchased from Thermo Fisher Scientific (Waltham, MA, USA), and β-glycerophosphate from Merck (Darmstadt, Germany). Fetal bovine serum was purchased from Thermo Scientific (Melbourne, VIC, Australia), and alizarin red S from Cosmo Bio (Tokyo, Japan).

### 4.2. Extraction, Solvent Fractionation, and Column Chromatography

The dried leaves of *E. polyandra* (495 g) were extracted three times using methanol (MeOH) in reflux for 3 h. After removing the solvent through evaporation, the extract (130 g) was dissolved in H_2_O and partitioned successively with *n*-hexane (3.2 g), ethyl acetate (EtOAc; 37.8 g), *n*-butanol (*n*-BuOH; 38.7 g), and H_2_O layers (38.0 g). After the bio-assays described below were performed with each fraction, the EtOAc-soluble fraction (4.2 g) was further separated using an ODS Column (2.8 × 37.5 cm i.d., CHROMATOREX ODS-DM1020T, FUJI SILYSIA CHEMICAL LTD., Tokyo, Japan) with the successive elution of H_2_O-MeOH (1:1; Fr.1; 1.8 g), H_2_O-MeOH (1:3; Fr.2; 0.6 g), MeOH (Fr.3; 1.19 g), MeOH-Acetone (1:1; Fr.4; 0.07 g), and Acetone (Fr.5; 0.03 g). Next, the Fr.1 (170 mg) was subjected to an MPLC column (Yamazen Ultra pack ODS-SM-50C, 37 × 300 mm i.d., Yamazen Corporation, Osaka, Japan) and eluted with H_2_O-Acetonitrile (5:2; with 0.1% formic acid) as the mobile phase at the flow rate of 10 mL/min to yield 18 sub-fractions. Using TLC analysis, compounds **1** (25.8 mg) and **2** (18.9 mg) were isolated from fraction No. 8 and No. 11, respectively. These compounds were then evaporated to remove the organic solvent. The resulting samples were stored at –20 °C before use.

### 4.3. Osteoblast Differentiation

Murine calvaria-derived osteoblastic MC3T3-E1 cells, kindly gifted from Dr. Yoshiyuki Hakeda (Department of Oral Anatomy, Meikai University School of Dentistry, Sakado, Saitama, Japan), were cultured in α-MEM containing 10% fetal bovine serum, 100 U/mL of penicillin, and 100 μg/mL of streptomycin at 37 °C in a humidified atmosphere of 5% CO_2_ in the air. The cells were seeded at a density of 1 × 10^4^/well in a 96-well plate or 2.5 × 10^4^/well in a 48-well plate (Corning; Corning, NY, USA) and cultured in the growth medium. After reaching subconfluency, the cells received the differentiation medium containing 100 μg/mL of ascorbic acid and 10 mM of β-glycerophosphate with or without the indicated test sample. The medium was changed every 3 or 4 days.

### 4.4. ALP Activity

The MC3T3-E1 cells were fixed with 4% paraformaldehyde (PFA), and then permeabilized with cold methanol and acetone (1:1) solution. The cells were stained with 2% NBT and 1% BCIP in 0.1 M Tris-buffered saline (pH 9.5) for 1 h at room temperature. To quantify the ALP activity, the staining materials were rinsed with PBS, dissolved using termination solution (20% acetic acid, 10% SDS, and 50% DMSO), and the absorbance was measured at 545 nm.

### 4.5. Cell Viability Analysis

The cell viability or cytotoxicity was assayed with MTT. The cells were incubated with 0.5 mg/mL MTT in medium for 3 h at 37 °C in 5% CO_2_. To quantify the cell viability, the formazan crystals in the cells were dissolved by the solution (50% DMSO and 50% 2-Propanol) and the absorbance was measured at 585 nm.

### 4.6. Mineralization Analysis

The calcium depositions of the differentiated MC3T3-E1 cells were assessed using alizarin red S staining. At the end of the culture, the cells were fixed with 4% buffered PFA, stained with 40 mM of alizarin red S (pH 6.4) for 20 min, and photographed.

### 4.7. Osteoclast Differentiation

The osteoclast precursor cell line, RAW264.7, kindly gifted from Dr. Takuya Sato (Department of Oral Anatomy, Meikai University School of Dentistry, Sakado, Saitama, Japan), was originally purchased from ATCC (Manassas, VA, USA). RAW264.7 cells were cultured in α-MEM containing 10% fetal bovine serum, 100 U/mL of penicillin, and 100 μg/mL of streptomycin at 37 °C in a humidified atmosphere of 5% CO_2_ in air. The cells were seeded at a density of 2 × 10^3^/well in a 96-well plate (Corning) and cultured in the growth medium. To induce osteoclastic differentiation, the cells were stimulated with 10 ng/mL RANKL with or without the indicated test sample and cultured for 3 days.

### 4.8. TRAP Activity Assay and Staining

For the TRAP activity, the culture medium (30 μL) was incubated with 30 μL of TRAP reagent (10 mM L of ascorbic acid, 40 mM of sodium tartrate dihydrate, 10 mM of disodium 4-nitrophenolphosphate, 0.2% Triton X-100, 4 mM of ethylenediaminetetraacetic acid, 40 mM of NaCl, 40 mM of sodium acetate buffer, pH 5.5) for 30 min at 37 °C. The reaction was stopped using 30 μL of 300 mM NaOH and the absorbance was measured at 405 nm.

For the TRAP staining, the cells were fixed with 4% PFA, the TRAP activity was stained using a leukocyte acid phosphatase kit (Sigma-Aldrich, St. Louis, MO, USA), according to the manufacturer’s instructions, and the cells were photographed.

### 4.9. RNA Extraction and Quantitative PCR (qPCR)

Total RNA was isolated with Isogen using a spin column kit (Nippon gene, Tokyo, Japan). First-strand cDNA was synthesized from total RNA using a ReverTra Ace (Toyobo, Osaka, Japan) according to the manufacturer’s instructions. qPCR was performed using the Taq Pro Universal SYBR-qPCR master Mix (Vazyme Biotech, Nanjing, China) on the Light Cycler system (Roche Diagnostics, Basel, Switzerland). Glyceraldehyde-3-phosphate dehydrogenase (*Gapdh*) was used as an internal control for the normalization of the gene expression level. The thermal cycling conditions were as follows: 95 °C for 10 min, followed by 40 cycles of 95 °C for 10 s, 60 °C for 10 s, and 72 °C for 15 s. The primers used to detect the genes of interest were as follows:

Osterix (*Osx*) 5′-AGCGACCACTTGAGCAAACAT-3′ and 5′-GCGGCTGATTGGCT TCTTCT-3′; *Alp*, 5′-TCTTGTCCGTGTCGCTCACCAT-3′ and 5′-CCAGAAAGACACCTTGACTGTGG-3′; *Opg*, 5′-CGGAAACAGAGAAGCCACGCAA-3′ and 5′-CTGTCCACCA AAACACTCAGCC-3′; *Acp5*, 5′-TTGCGACCATTGTTAGCCACATA-3′ and 5′-TCAGATC CATAGTGAAACCGCAAG-3′; *Ctsk*, 5′-CAGCAGAACGGAGGCATTGA-3′ and 5′-CTTTGCCGTGGCGTTATACATACA-3′; *Mmp9*, 5′-CCTGTGTGTTCCCGTTCATCT-3′ and 5′-CGCTGGAATGATCTAAGCCCA-3′; *Gapdh*, 5′-TGTGTCCGTCGTGGATCTGA-3′ and 5′-CCTGCTTCACCACCTTCTTGAT-3′.

The expression of each gene was calculated using the 2^−ΔΔCT^ methods. The gene expression ratio was shown as mean ±SD of triplicate determinations.

### 4.10. Instrumental Analysis

FAB-MS spectra were taken on a JMS-700 (2) (JEOL; Tokyo, Japan) using glycerol as a matrix. The data were analyzed using Delta NMR software (ver.5.0.5, JEOL). Then, 13C-NMR spectral data were recorded using a Varian 400-MR (Agilent; Santa Clara, CA, USA) spectrometer in DMSO-d6 using tetramethyl silane (TMS) as an internal standard. TLC was performed on a silica gel 60 RP-16 F254s plate (Merck).

### 4.11. Statistical Analysis

The in vitro bioassays were performed in three experiments. The values are expressed as the ratio of control (mean ± SD). Statistical analyses were performed utilizing one-way ANOVA followed by Bonferroni’s post hoc test. *p* < 0.05 was considered statistically significant.

## 5. Conclusions

We isolated osteogenically active compounds from the leaves of *E. polyandra*, namely, isoquercitrin and astragalin. These compounds, but also crude extracts of *E. polyandra,* promoted the differentiation of bone-forming osteoblasts and suppressed the differentiation of bone-resorbing osteoclasts. Therefore, *E. polyandra* may be a useful bioactive resource for the treatment of osteoporosis. Further evaluation using in vivo models may be required.

## Data Availability

All data generated or analyzed during this study are included in this article.

## Supplementary Materials

The following supporting information can be downloaded at https://www.mdpi.com/article/10.3390/ijms242417479/s1.
