# Supplementary material for: Isolation of Pro-Osteogenic Compounds from Euptelea polyandra That Reciprocally Regulate Osteoblast and Osteoclast Differentiation
Source: Int J Mol Sci. 2023 Dec 14;24(24):17479. doi: 10.3390/ijms242417479 (PMC10743613; doi:10.3390/ijms242417479)
Supplement: Supplementary file 1 [file ijms-24-17479-s001.zip › ijms-2691127-supplementary.pdf]

Supplementary

Isolation of pro-osteogenic compounds from *Euptelea polyandra* that reciprocally regulate osteoblast and osteoclast differentiation

Ryuichiro Suzuki [1,*], Yoshiaki Shirataki [1], Akito Tomomura [2], Kenjiro Bandow [2], Hiroshi Sakagami [3] and Mineko Tomomura [1,2,*]

[1] Department of Pharmaceutical Sciences, Faculty of Pharmacy and Pharmaceutical Sciences, Josai University, 1-1 Keyakidai, Sakado, Saitama, 350-0295 Japan

[2] Division of Biochemistry, Department of Oral Biology & Tissue Engineering, Meikai University School of Dentistry, Sakado, Saitama 350-0283, Japan.

[3] Meikai University Research Institute of Odontology (M-RIO), Sakado, Saitama 350-0283, Japan.

* Correspondence:

Mineko Tomomura: mtomomura@josai.ac.jp; mineko-t@dent.meikai.ac.jp

Ryuichiro Suzuki: ryu_suzu@josai.ac.jp

Contents

Scheme S1. Schematic representation of extraction and fractionation of *Euptelea polyandra*

Scheme S2. Schematic representation of isolation of compounds from *Euptelea polyandra*

File S1. Spectral data of isolated compounds from *Euptelea polyandra*

Scheme S1. Schematic representation of extraction and fractionation of *Euptelea polyandra*

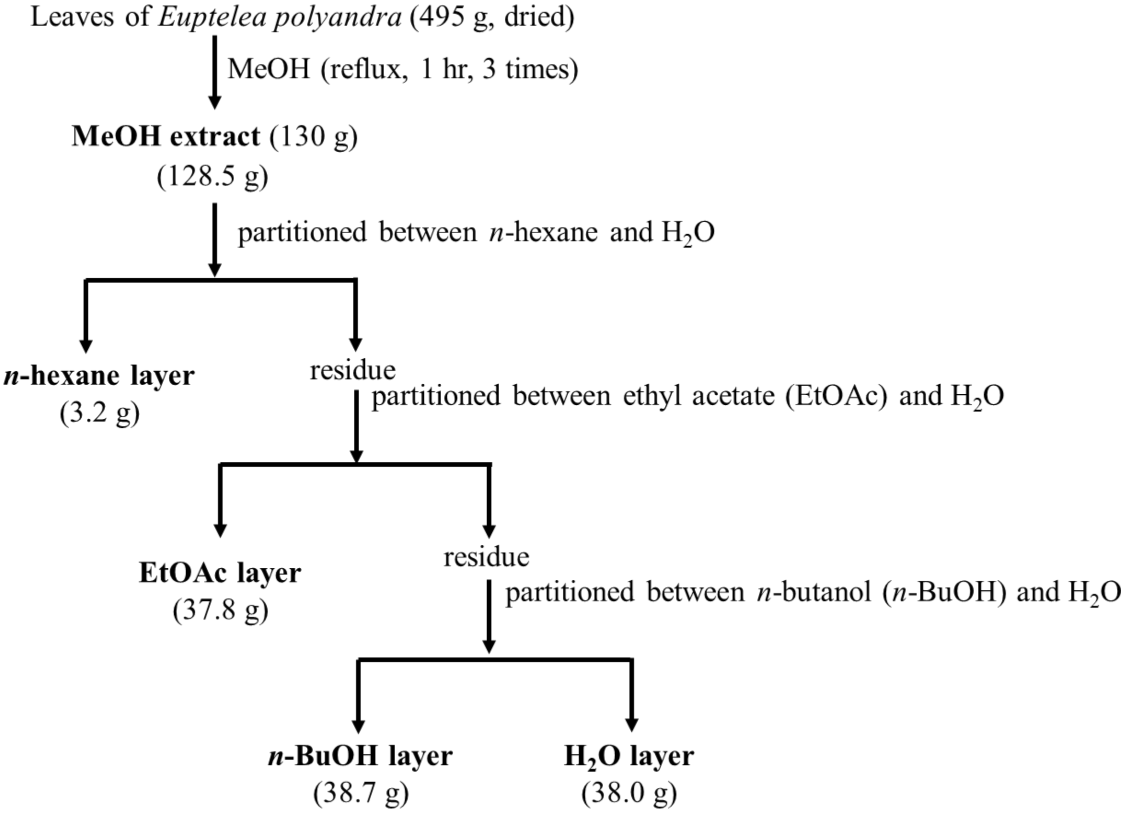

Scheme S2. Schematic representation of isolation of compounds from *Euptelea polyandra*

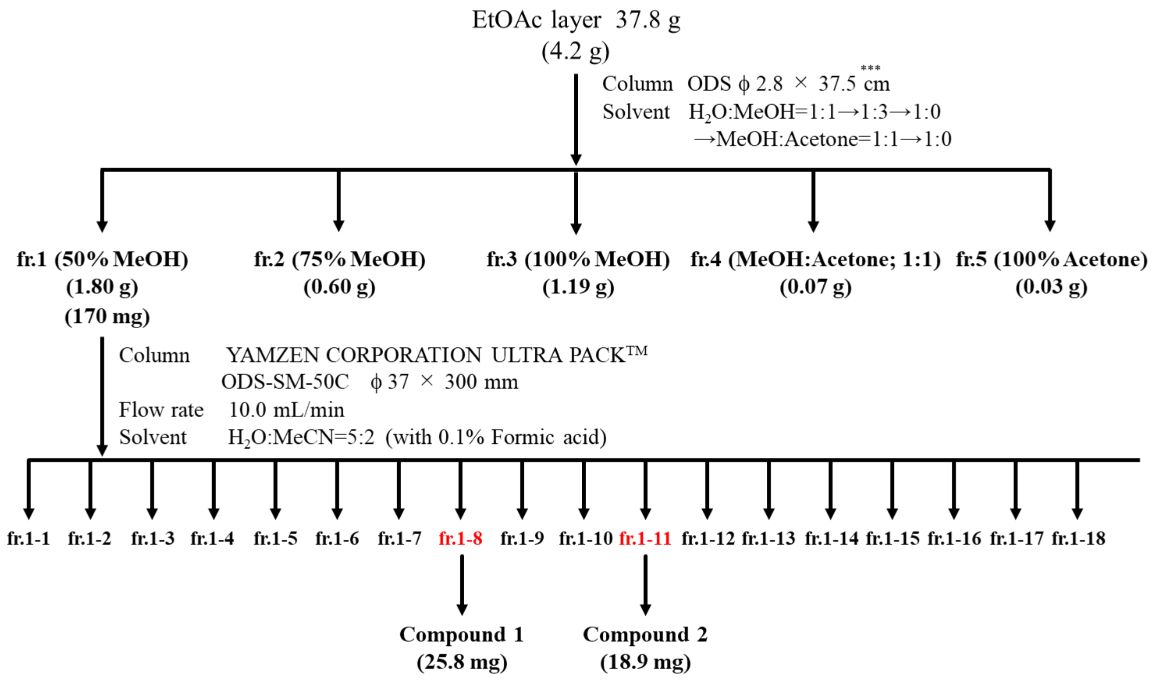

File S1. Spectral data of isolated compounds from *Euptelea polyandra*

Isoquercitrin (**1**): yellow powder, FAB-MS *m/z* 465 $[M+H]^+$, ^{13}C NMR (DMSO-d_6) δ_C:60.1 (C-6’’), 70.0 (C-4’’), 74.1 (C-2’’), 76.4 (C-3’’), 77.6 (C-5’’), 93.5 (C-8), 98.7 (C-6), 100.8 (C-1’’), 104.0 (C-10), 115.2 (C-2’), 116.2 (C-5’), 121.2 (C-1’), 121.6 (C-6’). 133.3 (C-3), 144.8 (C-3’), 148.5 (C-4’), 156.2 (C-2), 156.3 (C-9), 161.2 (C-5), 164.2 (C-7), 177.4 (C-4)

Astragalin (**2**): yellow powder, FAB-MS *m/z* 449 $[M+H]^+$, ^{13}C NMR (DMSO-d_6) δ_C:60.8 (C-6”), 69.9 (C-4”), 74.2 (C-2”), 76.4 (C-5”), 77.5 (C-3”), 93.7 (C-8), 98.8 (C-6), 100.9 (C-1”), 103.8 (C-10), 115.1 (C-3’, C-5’), 120,1 (C-1’), 130.8 (C-2’, C-6’), 133.1 (C-3), 156.1 (C-2), 156.4 (C-9), 160.0 (C-4’), 161.2 (C-5), 164.6 (C-7), 177.4 (C-4)
